# Meibum Expressibility Improvement as a Therapeutic Target of Intense Pulsed Light Treatment in Meibomian Gland Dysfunction and Its Association with Tear Inflammatory Cytokines

**DOI:** 10.1038/s41598-019-44000-0

**Published:** 2019-05-21

**Authors:** Moonjung Choi, Soo Jung Han, Yong Woo Ji, Young Joon Choi, Ikhyun Jun, Mutlaq Hamad Alotaibi, Byung Yi Ko, Eung Kweon Kim, Tae-im Kim, Sang Min Nam, Kyoung Yul Seo

**Affiliations:** 1 Department of Ophthalmology, Konyang University College of Medicine, Myunggok Medical Research Center, Daejeon, South Korea; 2 Institute of Vision Research, Department of Ophthalmology, Severance Hospital, Yonsei University College of Medicine, Seoul, Korea; 3 Department of Ophthalmology, National Health Insurance Service Ilsan Hospital, Goyang, South Korea; 4 Department of Ophthalmology, International St. Mary’s Hospital, Catholic Kwandong University College of Medicine, Incheon, South Korea; 5 Department of Ophthalmology, Prince Mohammad Bin Abdulaziz Hospital, Riyadh, Saudi Arabia; 6 Cornea Dystrophy Research Institute, Department of Ophthalmology, Severance Hospital, Yonsei University College of Medicine, Seoul, Korea; 7 Department of Ophthalmology, CHA Bundang Medical Center, CHA University, Seongnam, South Korea

**Keywords:** Interleukins, Corneal diseases

## Abstract

Many recent studies have demonstrated the efficacy of intense pulsed light (IPL) for the treatment of meibomian gland dysfunction (MGD); however, its effective treatment targets have not yet been elucidated. This study aimed to investigate the baseline characteristics associated with an improvement in symptoms after IPL treatment; to examine the course of change in inflammatory tear cytokines, meibomian gland function, and tear stability; and to investigate the correlation between cytokines and ocular surface parameters. Thirty participants underwent three sessions of IPL treatment. During each examination, tear film lipid layer interferometry, meibography, tear meniscus height measurement, tear sampling, and slit-lamp examination were performed, and the Ocular Surface Disease Index (OSDI) questionnaire was administered. Meibum quality, meibum expressibility, lid margin abnormality, tear film break-up time (TBUT), ocular surface staining, and the OSDI significantly improved after treatment. Poor meibum expressibility and short TBUT were associated with greater recovery in the OSDI after IPL. Tear levels of IL-4, IL-6, IL-10, IL-17A, and TNF-α decreased after IPL, and IL-6, and TNF-α were correlated with the improvement in meibum expressibility. Therefore, IPL treatment improved meibomian gland function, stabilized the tear film, and decreased ocular surface inflammation. Patients with obstructive MGD and tear instability were more likely to experience an improvement in ocular discomfort after IPL treatment.

## Introduction

Meibomian gland dysfunction (MGD) is a prevalent cause of evaporative dry eye, affecting more than 50% of the Asian population^1^. However, many patients do not benefit from currently available treatments such as lid hygiene, meibum expression, and anti-inflammatory therapy^2^.

Concurrent improvement of ocular surface conditions observed in patients treated for rosacea of their face, led to the potential implementation of intense pulsed light (IPL) for the treatment of MGD^3^. IPL has been widely used in dermatology to treat various conditions such as rosacea, benign vascular lesions, and pigmented lesions^4^. It utilizes a noncoherent, polychromatic light source to yield selective photothermolysis, wherein a specific wavelength of light is absorbed by a chromophore and converted into heat to destroy the target tissue^4,5^. Previous studies have reported favorable outcomes given the therapeutic effect of IPL in patients with MGD^6–14^. The potential mechanisms of action of IPL in MGD include the coagulation of telangiectasia, which may lead to a decrease in inflammation^15^, and the liquefaction of the viscous meibum and dilation of the clogged meibomian gland ducts caused by the heat energy^11^.

Tear cytokines have been reported to be elevated in dry eye and MGD, and to be correlated with symptoms and clinical parameters^16,17^. Therefore, a serial evaluation of the changes in tear cytokine levels and clinical assessment throughout the sequential treatment period would help explain the anti-inflammatory effect of IPL, as well as in determining the correlation between inflammation and the clinical outcome. A recent study showed that IPL treatment can reduce the levels of tear inflammatory markers IL-17A and IL-6 in patients with MGD^18^. In addition, the altered levels of IL-6 in tears correlated with the changes in the number of meibomian glands producing a clear secretion from the lower lid after IPL treatment^18^. Although IL-17A and IL-6 are well known to play a role in the pathogenesis of dry eye, MGD is a multifactorial disorder accompanied by ocular surface inflammation, but its etiology and pathogenesis remain unknown. TNF-α is another pleiotropic proinflammatory cytokine that has been associated with dry eye disease (DED)^19^. Moreover, DED has been considered a Th1-dominant disease; however, reports have suggested that autoantibodies may be involved in the pathogenic mechanism^20^. Therefore, it would be useful to also investigate cytokines associated with the Th2 response.

In the current study, the proposed anti-inflammatory effect of IPL was further investigated by analyzing additional tear inflammatory cytokines other than those previously evaluated. In addition, the clinical signs that changed in correlation with the inflammatory cytokine levels were investigated. The clinical characteristics of patients who experienced the greatest alleviation of ocular discomfort after IPL treatment were also explored. Hence, we sought to identify the important clinical factors and inflammatory cytokines associated with the treatment effect of IPL in patients with MGD.

## Results

### Clinical parameter changes and IPL

The mean scores (range) of meibum expressibility, lid margin abnormality, OSDI, and meibomian gland dropout were 2.9 (2–4), 3.5 (3–4), 58.2 (4.2–92.0), and 3.2 (2–5), respectively. The geometric means (range) of meibum quality score, ocular surface staining score, and TBUT were 3.4 (3–4), 1.8 (1–4), and 3.5 (0.5–9.0), respectively. Therefore, the subjects had moderate to severe MGD according to the clinical signs of MGD (meibum expressibility, lid margin abnormality, and meibomian gland dropout) and accompanying severe DED in terms of a short TBUT and high OSDI score.

**Figure 1:**
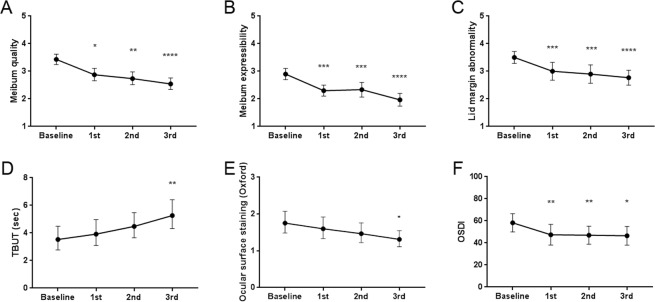
Change in clinical parameters following each intense pulsed light treatment session in patients with meibomian gland dysfunction. (**A**) Meibum quality, (**B**) meibum expressibility, (**C)** lid margin abnormality, (**D**) tear film break-up time (TBUT), (**E**) ocular surface staining score using the Oxford scheme, and (**F**) Ocular Surface Disease Index (OSDI). *P < 0.05; **P < 0.01; ***P < 0.001; ****P < 0.0001; Friedman test or repeated-measure analysis of variance (ANOVA) with post-hoc multiple comparison analysis, comparing the value after each session to that of the baseline. Individual points and error bars represent the mean (**B**,**C**,**F**) or the geometric mean (**A**,**D**,**E**) and 95% confidence interval.

The changes in clinical parameters over the time period of the three IPL sessions are outlined in Fig. 1. Meibum quality, meibum expressibility, and lid margin abnormality improved after IPL treatment, as shown by the decrease in their scores. TBUT increased and the ocular surface staining score decreased serially with treatment. The OSDI scores also decreased with IPL treatment. LLT, meibomian gland dropout, and tear meniscus area did not show any significant changes.

### Clinical parameters associated with the improvement in symptoms

We constructed a multiple regression model to identify the individual clinical parameters associated with the improvement in the discomfort score, OSDI, after IPL treatment (Table 1). The OSDI score after three treatment sessions decreased from the baseline score by 18.2 for each additional meibum expressibility score at baseline and increased from the baseline by 4.5 for each additional second of TBUT at baseline (Table 1). Thus, the worse baseline meibum expressibility scores and shorter baseline TBUT were associated with a greater reduction in the OSDI score after three treatment sessions. In addition, the OSDI score tended to decrease more in women than in men (Table 1).

**Table 1:** Multiple linear regression analysis of the association of the change in OSDI score with baseline clinical conditions.

Variable (Baseline value)	Unstandardized coefficient (B)	Standardized coefficient (β)	*P* value
Meibum expressibility	−18.2	−0.396	0.003
TBUT	4.5	0.453	0.007
Sex (female)	−19.1	−0.396	0.019

The change in the OSDI score is defined as the OSDI after the 3rd treatment session – the OSDI at baseline. Age (*P* = 0.950), meibum quality (*P* = 0.980), lid margin abnormality (*P* = 0.928), and ocular surface staining (*P* = 0.767) were excluded from the model by using the stepwise method.

*P* value for the overall model is 0.002 and adjusted R^2^ is 0.368.

OSDI = Ocular Surface Disease Index; TBUT = tear film break-up time.

### Tear inflammatory cytokines and IPL

**Figure 2:**
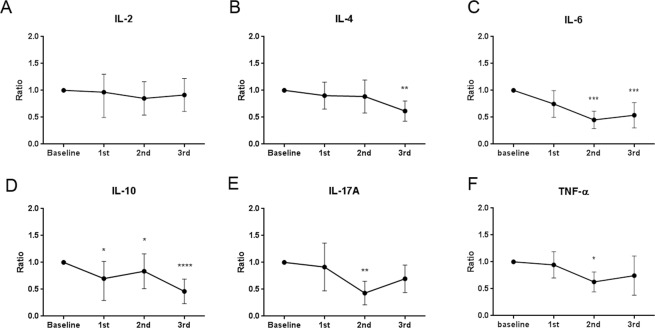
Change in cytokine profiles following each intense pulsed light (IPL) treatment session as a ratio compared to the baseline. *P < 0.05; **P < 0.01; ***P < 0.001; ****P < 0.0001; analysis of variance (ANOVA) (**C,F**) or Kruskal-Wallis test (**A**,**B**,**D**,**E**) with post-hoc analysis, comparing the value after each session to that of the baseline. Individual points and error bars represent the mean (**C**,**F**) or the median (**A**,**B**,**D**,**E**) and 95% confidence interval.

The tear cytokine levels were sequentially monitored to assess the anti-inflammatory effect of IPL. IL-4, IL-6, IL-10, IL-17A, and TNF-α levels showed a significant decrease over the time course (Fig. 2). However, the change in IL-2 level was not significant (*P* = 0.117, Kruskal-Wallis test). Although a slight increasing trend in IL-6, IL-17A, and TNF-α levels was observed after the third IPL session, the difference was not statistically significant (IL-6, *P* = 0.904; IL-17A, *P* = 0.394; TNF-α, *P* = 0.875).

### Correlation between meibomian gland function and tear inflammatory cytokines

**Figure 3:**
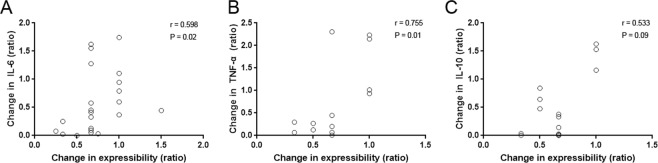
Scatter plot showing the correlations between the change in meibum expressibility and the change in the levels of tear cytokines IL-6 (**A**), TNF-α (**B**), and IL-10 (**C**). r = Spearman’s correlation coefficient.

To investigate whether the change in tear cytokine levels was related to the change in meibomian gland function, a correlation analysis was performed between the tear cytokine levels, IL-4, IL-6, IL-10, IL-17A and TNF-α, which decreased significantly after IPL treatment, and meibum quality and meibum expressibility. A positive correlation was observed between the changes in meibum expressibility and changes in IL-6 (r = 0.598, p = 0.02) and TNF-α (r = 0.755, p = 0.01) levels (Fig. 3). The correlation between the improvement in meibum expressibility and the decrease in IL-10 was significant in the correlation analysis (r = 0.533, p = 0.009), however became insignificant after the bonferroni correction (p = 0.09). Potential complications and adverse events, including uveitis and iris damage, did not occur in any of the patients.

## Discussion

IPL treatment was applied to patients with moderate to severe MGD and severe DED. Among the clinical parameters that responded to IPL treatment (Fig. 1), meibum expressibility showed the strongest relationship with the improvement in the OSDI score when other parameters were statistically controlled (Table 1). Additionally, meibum expressibility correlated with multiple inflammatory tear cytokines, IL-6 and TNF-α, whose levels were shown to decrease with IPL treatment (Figs 2 and 3).

IPL treatment improved the clinical parameters associated with meibomian gland function, including meibum quality, expressibility, and the lid margin abnormality score. A possible mechanism of action of IPL has been suggested as the local warming effect, which liquefies the inspissated meibum and encourages more regular outflow^11^. Previous studies have also reported improved meibum viscosity and oil flow score^10^ and a significant increase in meibomian gland expression^8,12,14^. The forced expression of meibomian glands after IPL treatment may also have contributed to the improvement in clinical parameters; however, previous studies have reported improved lipid layer grade^7^, improved meibomian gland secretion quality and expressibility^9^, increased TBUT^7,9^, and improvement in symptom scores^7,9^ after IPL treatment alone.

Improvement in the parameters related to meibomian gland function could improve the quality of the tear lipid layer and reinforce tear film integrity. In fact, TBUT and ocular surface staining improved after IPL treatment, and this result was well correlated with the results of previous studies^7,9,12–14^. However, the stabilization of the ocular surface was not accompanied by an increase either in the tear meniscus area or in LLT of the tear film. A previous study also found no change in tear meniscus height after IPL treatment^7^, and another study reported no change in LLT after IPL treatment^13^. Therefore, improved meibum quality and expressibility possibly strengthened the tear film and prevented tear evaporation, without affecting the tear volume itself.

Increased LLT was associated with increased age and female sex, as well as hypersecretory MGD and lid margin inflammation in a previous report^21^. Thus, the quantified LLT is affected by demographic factors and other confounders, and may not directly reflect meibomian gland function. A previous report that showed lipid layer improvement after IPL treatment examined the pattern of the tear film by using a different interferometry device^7^, while the interferometry in our study automatically calculated the LLT of the lower part of the cornea. Thus, the automatically quantified LLT may not be able to accurately portray the lipid layer status throughout the ocular surface, and the lipid quality of the tear film, instead of the thickness itself, may be more important in ocular surface stabilization. Qualitative lipid change is known to occur in MGD, and the change in lipid composition interferes with the adherence of the outermost lipid layer to the intermediate aqueous component, thus contributing to tear film instability and vulnerability to evaporation^22^.

The improvement in clinical parameters was accompanied by a reduction in patient-reported symptoms, the OSDI, and this was in concordance with the findings of previous studies^6–8,12,14^. The OSDI has been proven a reliable and valid tool in discriminating the severity of DED^23^. Women and those with a greater number of unexpressible meibomian glands and shorter TBUT showed a significant degree of reduction in the posttreatment OSDI score (Table 1). This was consistent with the findings of a previous study, which reported that patients with initially worse meibomian gland expressibility showed greater improvement on the OSDI after IPL treatment^12^. Therefore, those with obstructive MGD with decreased meibum expressibility and tear film instability are most likely to experience an improvement in ocular discomfort after IPL treatment.

Tear cytokine assays provide evidence for the ocular surface inflammation. IL-6 and IL-17A were reported to decrease in tears of MGD patients after IPL treatment^18^. In the current study, IL-4, IL-6, IL-10, IL-17A and TNF-α, which are cytokines known to be associated with DED^24,25^, decreased significantly following IPL treatment in MGD patients. The decrease in multiple tear cytokine concentration after IPL treatment may indicate that IPL was able to reduce inflammation which is one of the pathogenic mechanisms of MGD.

IL-6 and TNF-α are pleiotropic proinflammatory cytokines, which have been described as the key molecules in DED^19,24^. A previous report showed that IL-6 level was significantly increased in the tears of patients with DED, and it correlated with various ocular surface parameters, including TBUT, Schirmer test, and the keratoepithelioplasty score^26^. IL-17, whose level was also shown to be significantly increased in patients with MGD and DED^25^, is primarily produced by Th-17 cells and is known to increase the production of other inflammatory cytokines such as IL-6, TNF-α, IL-1, and IL-8, as well as the recruitment of leukocytes.

Although DED has been considered a Th1-dominant disease, evidence that autoantibodies may also be engaged in the pathogenic mechanism^20^ may explain our finding that the levels of IL-4 and IL-10, cytokines involved in the Th2 response, decreased after IPL treatment. IL-4 and IL-10 have previously been detected on the ocular surface of patients with DED^24^. IL-10 is predominantly an inhibitory cytokine that has multiple effects on immunoregulation, inflammation, and antibody production^27^. It is secreted not only by macrophages and Th2 cells, but also by regulatory T cells (Treg). Treg cells have been shown to suppress ocular surface inflammation associated with DED^28^, and studies have demonstrated that a Th17 cell subset, previously mentioned as a primary effector cell in DED, counteracts the Treg-mediated suppression in DED^29^.

The improvement in meibum expressibility positively correlated with the reduction in the levels of multiple tear inflammatory cytokines, such as IL-6 and TNF-α (Fig. 3). This finding was consistent with that of a previous report, which showed that the change in IL-6 level after IPL treatment correlated with the change in meibomian gland secretion in the lower lid^18^.

The probable mechanism behind the relationship between meibum expressibility and tear inflammatory cytokines could be explained by the following pathogenesis of MGD. Increased meibum viscosity and reduced expression may arise from the changes in meibum composition^30^. The stasis of the meibum can promote bacterial growth, which may then lead to the increased release of esterases and lipid-degrading lipases. The increased enzyme activity not only increases meibum melting temperature, but also generates free fatty acids that can lead to hyperkeratinization and inflammation^31^. These changes in lipid composition lead to further meibomian gland obstruction, ocular surface instability, and increased tear evaporation, contributing to the development of DED and patient discomfort. Therefore, ocular surface inflammation and meibomian gland expressibility are interactively involved in the cascade of the pathophysiologic mechanism of MGD, and our results suggest that the improvement in inflammation and meibum expressibility after IPL treatment is mutually inclusive, and that they might be important therapeutic targets of IPL treatment in patients with MGD.

The limitations of this study include a small sample size, lack of a control group, and a risk of the placebo effect and investigator bias. However, a previous paired-eye study showed that IPL treatment greatly improved tear film quality and reduced dry eye symptoms than did a placebo treatment^7^. The follow-up period after treatment termination was short. Hence, further investigation is needed to assess the long-term effectiveness and safety of IPL treatment. Future studies with larger sample sizes will be helpful in replicating and extending our findings. Additionally, it would have been helpful if the extent of lid margin vascularity was graded and its association with tear cytokines was evaluated, since one of the main mechanisms of action of IPL is coagulation of the superficial vessels. Furthermore, the clinical examinations were performed by one investigator, however we used the standardized grading scales reported by the International Workshop on Meibomian Gland Dysfunction^32^. Therefore, the measurement quality of the acquired data is less likely to be compromised.

## Conclusions

Patients with low meibum expressibility and tear film instability experienced greater improvement in symptoms after IPL treatment. The improvement in meibum expressibility was also associated with a decrease in tear inflammatory cytokine levels. Therefore, meibum expressibility improvement might be a good therapeutic target of IPL treatment in patients with MGD and DED, and could be an indicator of ocular surface inflammation during IPL treatment.

## Methods

### Patient selection

This prospective study adhered to the tenets of the Declaration of Helsinki and was approved by the Severance Hospital Institutional Review Board, Seoul, South Korea (1-2016-0010). All participants agreed and signed the written informed consent prior to enrollment. The study was registered at the Clinical Research Information Service (CRIS) under the registration number KCT0003051 (06/07/2018).

Participants over 19 years of age and diagnosed with moderate or severe MGD at Severance Hospital, Seoul, Korea, were screened for eligibility. MGD was staged according to the severity of symptoms, including ocular discomfort, and clinical signs, including lid margin features, meibum secretion, meibum expressibility, and ocular surface staining^2^. Participants were enrolled if they satisfied the following criteria for MGD staging. Moderate MGD was defined by moderate symptoms and MGD clinical signs (plugging and vascularity of the lid margins, meibum quality grade 2–3, and expressibility grade 3), and mild to moderate ocular surface staining (grade ≥ 1). Severe MGD was diagnosed on the basis of pronounced symptoms with limitation of daily activities, severe MGD signs (dropout, displacement of the lid margins, meibum quality grade ≥ 3, and expressibility grade 4), increased ocular surface staining (grade ≥ 1), and inflammatory signs (conjunctival hyperemia and phlyctenules). Patients with (1) Fitzpatrick skin type V or VI; (2) active allergy, infection, or ocular surface inflammatory disease unrelated to dry eye or MGD; (3) systemic diseases or medication use in which light therapy is contraindicated; (4) uncontrolled systemic disease; (5) ocular surgery history within 6 months before study initiation; (6) contact lens use; (7) tattoos, semipermanent makeup, and pigmented lesions in the treatment area; and (8) clinical skin treatments within 2 months were excluded, as were (9) pregnant patients and nursing mothers.

The eye with a higher stage of MGD was chosen for the study. If the MGD stage was equivalent in both the eyes, the right eye was enrolled. Thirty eyes of 30 patients who completed three sessions of IPL and four clinical examinations and tear sample collection were included in the analysis. The mean age of the patients was 51 ± 18 years, and 76.7% of them were women.

### Treatment technique

Patients received three sessions of IPL treatment at 3 week intervals. All treatment adhered to the Toyos protocol^6^. IPL-Aid Disposable Eye Shields (Honeywell Safety Products, Smithfield, RI, USA) were placed to protect the participants’ eyes. A cooling gel was generously applied to the treatment area, and homogenously sculpted light pulses of 590-nm wavelength and intensity ranging from 12 to 14 J/cm^2^ were delivered to the periocular skin inferior and lateral to the eye by using the M22 IPL machine (Lumenis Ltd., Israel). A 590-nm filter was selected to allow for selective photothermolysis of hemoglobin within the blood vessels, which had an optimal absorption range of 577–600 nm^33^. The fluence was initially set at 13 J/cm^2^, a previously reported setting for the treatment of rosacea and telangiectasia^34^, and was adjusted individually according to the patients’ tolerance and comfort. Approximately 15 overlapping pulses were applied from the preauricular area, across the cheeks and nose to the contralateral side, and bordering close to the inferior boundary of the eye shields to ensure the light pulses were delivered as close as possible to the lower eyelids. After the initial pass was completed, more ultrasound gel was applied, and the treatment was repeated for a second pass. After IPL treatment, manual expression of the meibomian glands of both the upper and lower eyelids was performed using meibum expressor forceps. The patients were instructed to maintain lid scrub and to use artificial tears during the treatment period.

### Clinical assessments

The clinical assessments were executed at baseline, at each separate treatment session, and at 3 weeks after the last session. All evaluations were performed before the IPL treatment at every visit. The order of the examinations was such that the influence of a preceding test on the subsequent test was minimized. All patients underwent tear film lipid layer interferometry, followed by tear meniscus area measurement by using anterior segment optical coherence tomography. Thereafter, tear sampling was performed, followed by slit-lamp examinations, including a fluorescein tear break-up time (TBUT), measurement of ocular surface staining, and examination of the lid margin and meibomian glands. All clinical examinations were undertaken by a single masked investigator (M.C.), and the IPL treatment was performed by another investigator (K.Y.S.).

Lipid layer thickness (LLT) measurement and meibography were performed using an interferometer (LipiView^®^, TearScience Inc, Morrisville, NC, USA) as previously described^35^. LLT is derived from the reflected tear film image, and is calculated as interferometric color units (ICUs), where 1 ICU represents approximately 1 nm. The maximum LLT that can be measured is 100 nm. The lower eyelids were everted to obtain infrared images of the meibomian glands. Meibomian gland dropout was scored on a 1 to 5 meiboscale, graded according to the area of gland loss (1, 0%; 2, < 25%; 3, 25–50%; 4, 51–75%; and 5, > 75%)^36^.

A 3-mm vertical image at the middle of the lower eyelids was scanned using Fourier-domain optical coherence tomography (RTVue; Optovue, Inc., Fremont, CA, USA) to measure the area for the lower tear meniscus^37^. It was defined as the area enclosed by the boundaries of the tear meniscus, cornea, and lower palpebral conjunctiva.

TBUT was measured by applying a fluorescein-impregnated strip (Haag-Streit, Koeniz, Switzerland) to the inferior palpebral conjunctiva. The mean time of three attempts was calculated. Thereafter, the Oxford scheme was used to grade corneal and conjunctival staining from 1 to 6^38^.

Firm digital pressure was applied to the five central glands of the lower lid to evaluate meibum expressibility and quality. Meibum expressibility was scored as 1, all 5 glands; 2, 3–4 glands; 3, 1–2 glands; and 4, 0 glands^39^, and meibum quality was scored as 1, clear; 2, cloudy; 3, cloudy and particulate; and 4, inspissated, and was recorded as the highest grade expressed by the examined glands^40^. The lid margin abnormality score was calculated as the sum of the following four parameters: vascular engorgement, meibomian gland orifice plugging, irregularity of the lid margin, and mucocutaneous junction displacement (each parameter was given 1 point if present)^41^. Subjective symptoms were assessed using the Ocular Surface Disease Index (OSDI)^23^.

### Tear sample collection and cytokine analysis

Tear samples were collected from all patients at every visit. We instilled 30 µL of phosphate-buffered saline into the inferior conjunctival fornix, and then collected 20 µL of the unstimulated tear fluid and buffer by using a micropipette. The samples were individually collected, each allocated into separate 0.5-mL Eppendorf tubes (Eppendorf, Fremont, CA, USA), and were preserved at a very low temperature of −70 °C until further analysis.

The levels of tear cytokines were analyzed using a multiplex bead-based immunoassay (BD^TM^ Cytometric Bead Array Human Soluble Protein Flex Set; BD Biosciences, San Jose, CA, USA). The cytokines IL-2, IL-4, IL-6, IL-10, IL-17A, and TNF-α were investigated. The tear samples were incubated with antibody-coated capture beads and the detector antibody-phycoerythrin agent at room temperature for 3 hours. The samples were washed to remove the unbound antibodies. Flow cytometry was performed, and FCAP Array^TM^ v3.0 (BD Cytometric Bead Array software; BD Biosciences) was used to interpolate the sample concentrations by comparison to a standard curve and to analyze the data.

### Statistical analysis

Prospective power calculations for sample size requirements were conducted before study initiation. A minimum of 25 participants was required to detect an effect size of 0.6 to achieve a power of 80% and a two-sided statistical significance level of 5%. To assess the time course changes in the clinical parameters over the three treatment sessions, repeated-measure analysis of variance (ANOVA) or Friedman test was applied depending on the normality of data. Those with normal distribution were analyzed using a parametric test, and the arithmetic mean was calculated. If the data were not normally distributed, logarithmic transformation of the dataset was performed, and the geometric mean was calculated. If significant differences were observed, the Bonferroni post-hoc test or Dunn’s test for multiple comparisons was performed to compare the baseline and posttreatment data at individual time points. Multiple regression models were constructed to identify baseline clinical parameters associated with the changes in the OSDI scores after treatment. The tear cytokine data were transformed into ratios by using the baseline value as the reference. The outliers were identified using robust regression followed by the outlier identification method and were excluded. Thereafter, ANOVA or the Kruskal-Wallis test was used to compare multiple variables, including the baseline and sequential measurements at each visit after the corresponding treatment session. If significant differences were observed, Dunnett’s or Dunn’s post-hoc test was performed to compare the baseline and posttreatment values after each session. Spearman’s rank-order correlation was used to investigate the association between the change in tear cytokine levels and the improvement in meibomian gland function, and Bonferroni correction was conducted. The data were transformed into ratios of the results after the final session to the baseline value, and these ratios were used in the analysis. Statistical analyses were conducted using IBM SPSS Statistics for Windows/Macintosh, Version 23.0 (IBM Corp., Armonk, NY, USA) and GraphPad Prism version 7.0 (GraphPad Software Inc., La Jolla, CA, USA).

## Data Availability

The datasets generated and/or analyzed during the current study are available from the corresponding author on reasonable request.

## References

[1] Alghamdi YA (2016). Epidemiology of Meibomian Gland Dysfunction in an Elderly Population. Cornea.

[2] Nichols KK (2011). The international workshop on meibomian gland dysfunction: executive summary. Invest Ophthalmol Vis Sci.

[3] Toys R, Buffa, C., Youngerman, S. *Case report: Dry-eye symptoms improve with intense pulsed light treatment* (2005).

[4] Raulin C, Greve B, Grema H (2003). IPL technology: a review. Lasers Surg Med.

[5] Babilas P, Schreml S, Szeimies RM, Landthaler M (2010). Intense pulsed light (IPL): a review. Lasers Surg Med.

[6] Toyos R, McGill W, Briscoe D (2015). Intense Pulsed Light Treatment for Dry Eye Disease Due to Meibomian Gland Dysfunction; A 3-Year Retrospective Study. Photomed Laser Surg.

[7] Craig JP, Chen YH, Turnbull PR (2015). Prospective trial of intense pulsed light for the treatment of meibomian gland dysfunction. Invest Ophthalmol Vis Sci.

[8] Vegunta S, Patel D, Shen JF (2016). Combination Therapy of Intense Pulsed Light Therapy and Meibomian Gland Expression (IPL/MGX) Can Improve Dry Eye Symptoms and Meibomian Gland Function in Patients With Refractory Dry Eye: A Retrospective Analysis. Cornea.

[9] Jiang X (2016). Evaluation of the Safety and Effectiveness of Intense Pulsed Light in the Treatment of Meibomian Gland Dysfunction. J Ophthalmol.

[10] Gupta PK, Vora GK, Matossian C, Kim M, Stinnett S (2016). Outcomes of intense pulsed light therapy for treatment of evaporative dry eye disease. Can J Ophthalmol.

[11] Vora GK, Gupta PK (2015). Intense pulsed light therapy for the treatment of evaporative dry eye disease. Curr Opin Ophthalmol.

[12] Albietz JM, Schmid KL (2018). Intense pulsed light treatment and meibomian gland expression for moderate to advanced meibomian gland dysfunction. Clin Exp Optom.

[13] Dell SJ, Gaster RN, Barbarino SC, Cunningham DN (2017). Prospective evaluation of intense pulsed light and meibomian gland expression efficacy on relieving signs and symptoms of dry eye disease due to meibomian gland dysfunction. Clin Ophthalmol.

[14] Yin Y, Liu N, Gong L, Song N (2018). Changes in the Meibomian Gland After Exposure to Intense Pulsed Light in Meibomian Gland Dysfunction (MGD) Patients. Curr Eye Res.

[15] Papageorgiou P, Clayton W, Norwood S, Chopra S, Rustin M (2008). Treatment of rosacea with intense pulsed light: significant improvement and long-lasting results. Br J Dermatol.

[16] Enriquez-de-Salamanca A (2010). Tear cytokine and chemokine analysis and clinical correlations in evaporative-type dry eye disease. Molecular vision.

[17] Lam H (2009). Tear cytokine profiles in dysfunctional tear syndrome. Am J Ophthalmol.

[18] Liu R (2017). Analysis of Cytokine Levels in Tears and Clinical Correlations After Intense Pulsed Light Treating Meibomian Gland Dysfunction. Am J Ophthalmol.

[19] Stevenson W, Chauhan SK, Dana R (2012). Dry eye disease: an immune-mediated ocular surface disorder. Arch Ophthalmol.

[20] Stern ME, Schaumburg CS, Pflugfelder SC (2013). Dry eye as a mucosal autoimmune disease. International reviews of immunology.

[21] Jung JW (2016). Analysis of Factors Associated With the Tear Film Lipid Layer Thickness in Normal Eyes and Patients With Dry Eye Syndrome. Invest Ophthalmol Vis Sci.

[22] Knop E, Knop N, Millar T, Obata H, Sullivan DA (2011). The international workshop on meibomian gland dysfunction: report of the subcommittee on anatomy, physiology, and pathophysiology of the meibomian gland. Invest Ophthalmol Vis Sci.

[23] Schiffman RM, Christianson MD, Jacobsen G, Hirsch JD, Reis BL (2000). Reliability and validity of the Ocular Surface Disease Index. Arch Ophthalmol.

[24] Massingale ML (2009). Analysis of inflammatory cytokines in the tears of dry eye patients. Cornea.

[25] Kang MH (2011). Interleukin-17 in various ocular surface inflammatory diseases. Journal of Korean medical science.

[26] Yoon KC, Jeong IY, Park YG, Yang SY (2007). Interleukin-6 and tumor necrosis factor-alpha levels in tears of patients with dry eye syndrome. Cornea.

[27] Iyer SS, Cheng G (2012). Role of interleukin 10 transcriptional regulation in inflammation and autoimmune disease. Crit Rev Immunol.

[28] Siemasko KF (2008). *In vitro* expanded CD4 + CD25 + Foxp3 + regulatory T cells maintain a normal phenotype and suppress immune-mediated ocular surface inflammation. Invest Ophthalmol Vis Sci.

[29] Chauhan SK (2009). Autoimmunity in dry eye is due to resistance of Th17 to Treg suppression. Journal of immunology.

[30] Nelson JD (2011). The international workshop on meibomian gland dysfunction: report of the definition and classification subcommittee. Invest Ophthalmol Vis Sci.

[31] Baudouin C (2016). Revisiting the vicious circle of dry eye disease: a focus on the pathophysiology of meibomian gland dysfunction. The British journal of ophthalmology.

[32] Tomlinson A (2011). The international workshop on meibomian gland dysfunction: report of the diagnosis subcommittee. Invest Ophthalmol Vis Sci.

[33] Srinivas CR, Kumaresan M (2011). Lasers for vascular lesions: standard guidelines of care. Indian J Dermatol Venereol Leprol.

[34] Kassir R, Kolluru A, Kassir M (2011). Intense pulsed light for the treatment of rosacea and telangiectasias. J Cosmet Laser Ther.

[35] Eom Y, Lee JS, Kang SY, Kim HM, Song JS (2013). Correlation between quantitative measurements of tear film lipid layer thickness and meibomian gland loss in patients with obstructive meibomian gland dysfunction and normal controls. Am J Ophthalmol.

[36] Pult H, Riede-Pult B (2013). Comparison of subjective grading and objective assessment in meibography. Cont Lens Anterior Eye.

[37] Han KE (2014). Evaluation of dry eye and meibomian gland dysfunction after cataract surgery. Am J Ophthalmol.

[38] Bron AJ, Evans VE, Smith JA (2003). Grading of corneal and conjunctival staining in the context of other dry eye tests. Cornea.

[39] Pflugfelder SC (1998). Evaluation of subjective assessments and objective diagnostic tests for diagnosing tear-film disorders known to cause ocular irritation. Cornea.

[40] Bron AJ, Benjamin L, Snibson GR (1991). Meibomian gland disease. Classification and grading of lid changes. Eye (Lond).

[41] Arita R (2009). Proposed diagnostic criteria for obstructive meibomian gland dysfunction. Ophthalmology.

